# Bilateral ulnar longitudinal deficiency with oligodactyly in newborn

**DOI:** 10.1590/1984-0462/2025/43/2024101

**Published:** 2024-11-29

**Authors:** Sofia Cruzes Moysés Simão, Júlia Avelans Pires da Silva, Ariel Ortega Miranda, Rayza de Sousa Costa, Carolina Schlindwein Mariano Ferreira, Regina Yumi Saito

**Affiliations:** a Universidade Nove de Julho, São Paulo, SP, Brazil.; b Hospital Ipiranga, São Paulo, SP, Brazil.; c Hospital do Servidor Público Municipal, São Paulo, SP, Brazil.

**Keywords:** Malformations, Upper limbs, Hemimelia, Genetics, Hand deformities, Malformação, Membros superiores, Hemimelia, Genética, Deformidades da mão

## Abstract

**Objective:**

The objective of this study was to report a case of bilateral ulnar longitudinal deficiency with oligodactyly in a male newborn.

**Case description:**

A full-term male newborn, born following an uncomplicated gestation with no abnormalities detected on prenatal ultrasounds, presented upper limb malformations described as shortening of the left forearm and absence of three digits bilaterally upon neonatal physical examination. Diagnostic investigations including X-rays, abdominal ultrasound, head ultrasound, echocardiogram, and karyotype analysis were conducted, facilitating detailed identification of the malformations and exclusion of other anomalies, thereby suggesting the diagnosis of congenital longitudinal deficiency of the ulna. Discharge planning encompassed supportive care and rehabilitation as per the patient’s needs.

**Comments:**

Ulnar longitudinal deficiency is a rare congenital upper limb malformation, whose estimated incidence is 1:100,000 newborns. It is believed to be related to the Sonic Hedgehog gene, and the upper limb anomalies vary according to the ulnar involvement. The early diagnosis is not routine, being more common at the first physical examination with the aid of imaging tests.

## RESUMO

**Objetivo:**

Relatar um caso de deficiência longitudinal congênita da ulna com oligodactilia bilateral em recém-nascido do sexo masculino.

**Descrição do caso:**

Recém-nascido do sexo masculino nascido a termo, cuja gestação foi normal e sem alterações nas ultrassonografias durante o pré-natal, apresentou malformações em membros superiores, com encurtamento do antebraço esquerdo e ausência de três quirodáctilos bilateralmente durante o exame físico neonatal. Foi realizado um estudo diagnóstico baseado em exames complementares como raio X, ultrassonografia abdominal e transfontanela, ecocardiograma e cariótipo, os quais permitiram a identificação detalhada das malformações e a exclusão de demais anomalias, definindo o diagnóstico de deficiência longitudinal congênita da ulna. As condutas da alta hospitalar foram baseadas na necessidade de suporte e reabilitação para o paciente.

**Comentários:**

A deficiência longitudinal congênita da ulna é uma rara malformação de membros superiores, cuja incidência estimada é de 1:100.000 recém-nascidos. Acredita-se que esteja relacionada com o gene Sonic Hedgehog, e as anomalias presentes nos membros superiores variam de acordo com o acometimento ulnar. O diagnóstico precoce não é rotineiro, sendo mais comum ao primeiro exame físico do recém-nascido, com o auxílio de exames de imagem.

## INTRODUCTION

Ulnar longitudinal deficiency (ULD) is a rare and sporadic congenital malformation of the upper limbs, characterized by partial or complete ulnar absence. Also known as “ulnar club hand,” “congenital ulnar hemimelia,” or “postaxial longitudinal deficiency of the upper limb,” the ULD is probably related to an interruption of the Sonic Hedgehog (SHH) gene, responsible for the ulnar-sided forearm structure formation, thought to occur during weeks 4 and 5 of fetal development.^1,2^

Usually, ULD is an isolated condition and is rarely associated with internal organs malformations or systemic syndromes. Although other skeletal malformations are frequently seen, such as congenital short femur, phocomelia, fibular hemimelia, congenital scoliosis, and digital abnormalities. The patient’s hand is normally hypoplastic, and 90% of cases have missing digits, but other conditions like syndactyly, thumb abnormalities, rotated metacarpals, and hypoplastic tendons and muscles can appear.^3,4^

In addition to the clinical findings, the diagnosis might be confirmed with a radiological study of the affected body parts. There are two relevant classifications for ulnar longitudinal deficiency: the modified Bayne’s system to describe forearm and elbow deformities, and, for hand ones, the most accepted classification is from Cole and Manske.^3,5^ The treatment can be surgical or conservative, depending on how severe the involvement of the arm is, including the elbow and forearm anomalies classification, as well as hand position and presence of digital abnormalities.^6^

## CASE REPORT

A male newborn was delivered by cesarean section at 40 weeks due to fetal distress. Mother was 25 years old and had three other children, all healthy and without any abnormalities. Mother was up to date on vaccinations and took iron and folic acid supplements during the current pregnancy. She denied addictions or a family history of malformation. She attended eight prenatal visits without complications, with all her serologies negative, no risk of maternal-fetal blood incompatibility, and no abnormalities found on the morphological ultrasound.

**Figure 1:**
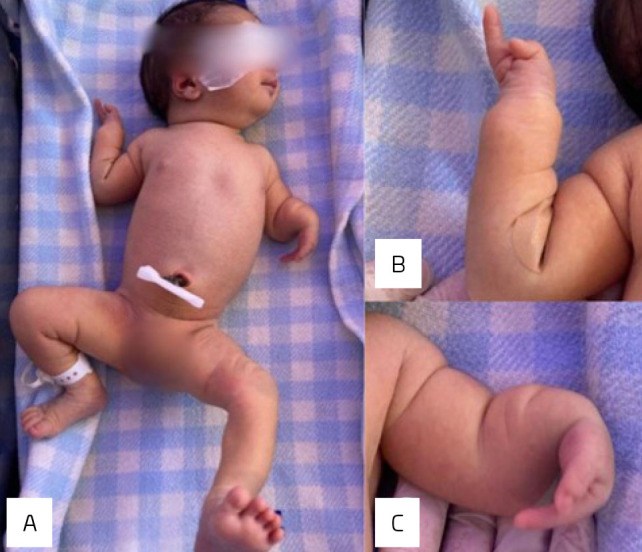
(A) Newborn with upper limbs malformations. (B) Right upper limb. Forearm with preserved flexion and absence of three fingers on the right hand. (C) Left upper limb showing shortening and rotation of the left forearm, ulnar deviation of the left hand, and the absence of three fingers. These malformations demonstrate ULD’s characteristics and exhibit one of the most common dysplasias of the ulna: the Ulnar Club Hand.

Through neonatal physical examination at birth, the neonatologist noticed malformation of both hands and left forearm, described as absence of three fingers on both hands and shortening of the left forearm (Figure 1). Further investigation with X-rays confirmed left ulnar absence along with a deformed radius, dislocation of the elbow joint, and bilateral absence of the third, fourth, and fifth digits and metacarpi. Karyotype, echocardiogram, abdominal and head ultrasounds, and computed tomography (CT) of the head and spine were normal.

The set of clinical findings and radiological results found in the case determined the diagnosis of ulnar longitudinal deficiency, and, after 4 days of care, the mother and the infant were discharged. As management, the newborn was referred to a neurologist, a geneticist, and an orthopedist for follow-up, showing expected development for his age currently.

## DISCUSSION

Ulnar longitudinal deficiency (ULD) is one of the rarest malformations of the upper limbs, which has the radial longitudinal deficiency (RLD) as the most common deficiency. ULD has an estimated incidence of 1:100,000 newborns, four to ten times less usual than RLD. Previously known as ulnar club hand, this condition is marked by underdevelopment or absence of the ulna bone. Common clinical findings during a physical exam are shortening of the forearm and ulnar deviation of the hand. ULD is generally isolated, and systemic findings are unusual. However, it can be associated with other skeletal malformations, such as digital abnormalities.^3,7,8^

Congenital limb defects can be divided into two categories: transverse and longitudinal defects. Longitudinal limb deficiencies refer to hypoplasia or the complete absence of a bone — or several bones — parallel to the axis of the limb. Radial and Ulnar Longitudinal deficiencies are the most common types of congenital limb defects.^7,9^ ULD represents a set of abnormalities that affect the ulnar margin of the upper extremity, in which upper arm and forearm deformities follow the ulnar longitudinal axis, although structures of the radial margin can be affected as well.^8^ About 70% of the ULD presentations are unilateral, mostly right-sided, and incomplete, with a common presentation including a shortened forearm, ulnar-sided hand deviation, radial head subluxation, and fixed flexion deformity of the elbow joint.^1,7^ Usually, the hand is hypoplastic, and about 90% of the patients have absent fingers.^3^

The etiologies of congenital limb malformations are frequently related to the Sonic Hedgehog (SHH) gene pathway, responsible for the anteroposterior developmental axis. Embryologically, disrupting the production of SHH from the zone of polarizing activity posteriorizes the developing limb, and the SHH deficiency explains the finger anomalies that may occur in cases of ULD.^2^ Since the SHH is also responsible for developing four ulnar-sided digits, its lack of function causes digit loss.^10^

ULD can appear isolated but can also be associated with other skeletal malformations.^4^ Differential diagnoses include Holt-Oram syndrome, Aase-Smith syndrome, Thrombocytopenia and absent radius syndrome (TAR), Levy-Hollister syndrome, Weyers ulnar ray-oligodactyly, Femur-fibula-ulna complex, Ulnar hypoplasia-split foot syndrome, Cornelia de Lange Syndrome, and Ulnar-mammary syndrome (Table 1).^11-18^ However, due to the predominantly syndromic nature of these pathologies, they can be ruled out in the reported patient’s case. Genetic counseling and molecular genetic testing could be useful for excluding these syndromes, though the ULD’s diagnosis is mostly clinical and confirmed by a radiological study.^10^

**Table 1:** Differential diagnosis of ulnar hemimelia: musculoskeletal characteristics and associated defects.

Malformation	Musculoskeletal	Associated defects
Holt-Oram syndrome	Abnormalities in the first digit of the hand such as triphalangeal, hypoplasia, aplasia, or even thumbs on the same plane as the other fingers (finger-like thumbs).	Congenital heart disease: atrial septal defect (ostium secundum is present in 34% of the cases); atrioventricular conduction disorders; vascular hypoplasia.
Aase-Smith syndrome	Triphalangeal thumbs; mild radial hypoplasia; narrow shoulders; delayed closure of the fontanelles.	Congenital heart disease: atrial septal defect (ostium secundum is present in 34% of the cases); atrioventricular conduction disorders; vascular hypoplasia.
Thrombocytopenia and absent radius syndrome (TAR)	Bilateral radial agenesis with preservation of the thumb.	Thrombocytopenia; cardiac, craniofacial, digestive, urogenital, and psychiatric malformations; intolerance to cow’s milk.
Levy-Hollister syndrome	Dental and skeletal anomalies, particularly in the hands and feet (clinodactyly and thumb hypoplasia are the most common findings).	Hypoplasia, aplasia, or atresia of the lacrimal and/or salivary systems; malformations of the external ear with or without hearing loss.
Weyers ulnar ray-oligodactyly	Ulnar or radial defect; ectrodactyly. Fibular reduction.	Heart, splenic, and renal abnormalities; single central incisor; abnormality of head and neck (cleft and high palate, cleft upper lip, and narrow face).
Femur-fibula-ulna complex	Defects of the femur, fibula, and ulna/ulnar rays; hypoplasia/aplasia of postaxial elements; peromelia of the humerus; humeroradial synostosis. Hypoplasia/aplasia of the postaxial; proximal femoral defect.	
Ulnar hypoplasia-split foot syndrome	Shortened ulna or complete ulnar absence; curved and thickened radius; the absence of the fifth metacarpal and phalange. Lobster-claw architecture of the foot.	
Cornelia de Lange Syndrome	Absent forearm; radioulnar synostosis; absent radius or ulna; oligodactyly.	Facial features (shortened nasal bridge, anteverted nares, synophrys, arched and thick eyebrows, synophrys, microcephaly; growth restriction, hypertrichosis).
Ulnar-mammary syndrome	Hypoplasia of the terminal phalanx of the fifth digit; ulna and/or radius; hand hypoplasia or aplasia.	Hypoplasia of mammary glands and apocrine glands; abnormal development of teeth, palate, and genitalia; obesity.

The early diagnosis of ULD and other upper limb differences is not usual. Even though fetal ultrasound is a crucial tool for prenatal diagnosis of fetal anomalies, studies show that the overall sensitivity for detecting them is low.^19,20^ Therefore, ULD is usually suspected by birth at the first physical examination. The main exam to confirm the diagnosis of ULD is the X-ray of the arm, forearm, and hand, where it will be possible to observe any anomalies present, including malformations or even the absence of one or more bones. There are two classifications used to differentiate the types of ULD: Bayne’s classification and Cole and Manske’s classification.^2,3,21^

**Figure 2:**
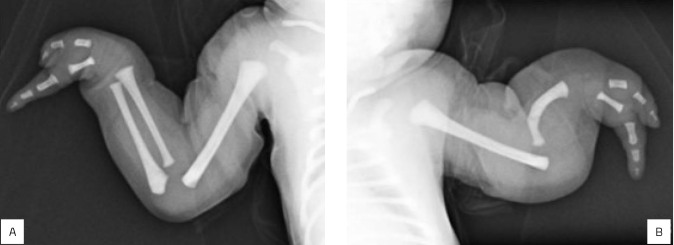
(A) X-ray of the right upper limb corresponding to Bayne’s classification 1 and Cole and Manske’s classification C. (B) X-ray of the left upper limb corresponding to Bayne’s classification 4 and Cole and Manske’s classification C.

Bayne’s classification divides ULD into four different types: type I is the ulnar hypoplasia with normal epiphysis, type II has partial ulnar aplasia, and this is the most common type of ULD. Type III corresponds to complete ulnar absence plus carpal and digital anomalies. Type IV is related to radiohumeral synostosis.^2,22^ On the contrary, Cole and Manske’s classification is divided into six types, in which type 0 represents a normal ulna with hand anomalies, type I hypoplastic ulna with the presence of both epiphyses, type II distal ulnar aplasia, type III complete ulnar aplasia, type IV radiohumeral synostosis, and type V proximal ULD.^21^ In the reported case, the left upper limb corresponds to Bayne’s classification IV and Cole and Manske’s classification C. Meanwhile, the right upper limb corresponds to Bayne’s classification 1 and Cole and Manske’s classification C (Figure 2).

Managing ULD cases can be complex and should be highly individualized. Operative or nonsurgical approaches could be chosen, depending on how severe the ulnar involvement is and the classification of the elbow and arm anomalies. Therefore, management includes referral to a geneticist, orthopedic, and plastic surgeon.^8^ Surgical intervention is employed as necessary to enhance limb functionality. In cases involving the hand, corrective procedures for syndactyly and reconstruction of the first commissure are conducted if required. For forearm issues such as hyperpronation or angular deformities, radial osteotomy is considered. In instances of distal ulnar absence, the option of constructing a single bone may arise. However, caution is advised against dissecting the radial head, as it could potentially destabilize the elbow.^3^

## Data Availability

The database that originated the article is available with the corresponding author.
